# Interlayer Connectivity Affects the Coherence Resonance and Population Activity Patterns in Two-Layered Networks of Excitatory and Inhibitory Neurons

**DOI:** 10.3389/fncom.2022.885720

**Published:** 2022-04-18

**Authors:** David Ristič, Marko Gosak

**Affiliations:** ^1^Faculty of Natural Sciences and Mathematics, University of Maribor, Maribor, Slovenia; ^2^Faculty of Medicine, University of Maribor, Maribor, Slovenia

**Keywords:** neuronal dynamics, coherence resonance, excitatory neurons, inhibitory neurons, neural network, multilayer network, interlayer connectivity

## Abstract

The firing patterns of neuronal populations often exhibit emergent collective oscillations, which can display substantial regularity even though the dynamics of individual elements is very stochastic. One of the many phenomena that is often studied in this context is coherence resonance, where additional noise leads to improved regularity of spiking activity in neurons. In this work, we investigate how the coherence resonance phenomenon manifests itself in populations of excitatory and inhibitory neurons. In our simulations, we use the coupled FitzHugh-Nagumo oscillators in the excitable regime and in the presence of neuronal noise. Formally, our model is based on the concept of a two-layered network, where one layer contains inhibitory neurons, the other excitatory neurons, and the interlayer connections represent heterotypic interactions. The neuronal activity is simulated in realistic coupling schemes in which neurons within each layer are connected with undirected connections, whereas neurons of different types are connected with directed interlayer connections. In this setting, we investigate how different neurophysiological determinants affect the coherence resonance. Specifically, we focus on the proportion of inhibitory neurons, the proportion of excitatory interlayer axons, and the architecture of interlayer connections between inhibitory and excitatory neurons. Our results reveal that the regularity of simulated neural activity can be increased by a stronger damping of the excitatory layer. This can be accomplished with a higher proportion of inhibitory neurons, a higher fraction of inhibitory interlayer axons, a stronger coupling between inhibitory axons, or by a heterogeneous configuration of interlayer connections. Our approach of modeling multilayered neuronal networks in combination with stochastic dynamics offers a novel perspective on how the neural architecture can affect neural information processing and provide possible applications in designing networks of artificial neural circuits to optimize their function *via* noise-induced phenomena.

## Introduction

Ensembles of neurons demonstrate a rich variety of coherent dynamics at the macroscopic scale which results from the input of multiple oscillatory signals and random perturbations at the microscopic scale. How neuronal populations adjust their dynamical responses to the superposition of these local noisy signals is of fundamental importance for information processing and plays a vital role in a variety of cognitive, motoric, and linguistic tasks (Riehle et al., 1997; Oram et al., 1999; Horwitz and Braun, 2004). Importantly, neuronal activity does not only depend on intrinsic neuronal properties and network architecture but is also heavily influenced by random fluctuations (Stein et al., 2005; Chialvo, 2010). Neuronal noise is a natural and unavoidable factor that originates from random opening and closing of ionic channels, stochastic nature of neuronal mechanisms, and noisy biochemical processes that underlie synaptic transmission (Faisal et al., 2008; Guo et al., 2018). While in most of the systems noise is mostly an undesirable component, it is now widely accepted that its presence is crucial to the proper functioning of neurons and can even enhance information processing capabilities and the regularity of neuronal activity (Lindner et al., 2004; McDonnell and Ward, 2011; Guo et al., 2018). This has led to a tremendous interest in investigating the sources and impact of intrinsic fluctuations in the nervous system and stems from the advances in experimental methods for identifying it as well as from a growing body of computational works demonstrating its functional consequences.

Neuronal noise has been shown to give rise to various collective dynamical behaviors, such as stochastic synchronization (Yang and Cao, 2009; Zakharova et al., 2013) or the induction of stochastic bifurcations (Hänggi and Bartussek, 1996; Gosak et al., 2008; Zakharova et al., 2010) and chimera states (Semenova et al., 2016; Majhi et al., 2019). Perhaps, the most famous examples of the so-called stochastic facilitation in neuronal systems are stochastic and coherence resonance. The former refers to the scenario where an appropriate intensity of noise evokes the best correlation between a weak periodic deterministic stimulus and the system's response (Longtin, 1993; McDonnell and Abbott, 2009; Calim et al., 2021), whereas the later encompasses a noise-induced enhancement in regularity of excited oscillations without external driving (Pikovsky and Kurths, 1997; Lee et al., 1998). Coherence is significant for communication within the brain (Deco and Kringelbach, 2016), and coherence resonance was recently suggested as a mechanism for improving neural communication (Pisarchik et al., 2019) and visual information processing (Itzcovich et al., 2017). Most importantly, this phenomenon is not only physiologically important but also theoretically appealing and challenging to understand. As a result, numerous computational models have been developed to elucidate the underlying mechanisms (Pradines et al., 1999; Ushakov et al., 2005; Beggs and Timme, 2012; Yu et al., 2017a; Guan et al., 2020; Baspinar et al., 2021). While primal investigations of coherence resonance in neuronal systems have scrutinized mainly systems with relatively small numbers of degrees of freedom, the scope has been shifting to coupled and spatially extended systems with many degrees of freedom (Lindner et al., 2004; Sagués et al., 2007; Kim et al., 2015), particularly to such complex networks describing connections between the individual units. Specifically, previous endeavors have revealed that the coherence resonance phenomenon can be modulated by the network size (Toral et al., 2003; Wang et al., 2005), as well as by the structure of the underlying network (Kwon and Moon, 2002; Gosak et al., 2010). Moreover, incorporating realistic neurophysiological features into neuronal network models, such as heterogeneity, spike-timing-dependent plasticity, and information transmission delays, was found to crucially affect the coherence resonance and can lead to very interesting dynamical behavior (Ozer and Uzuntarla, 2008; Li et al., 2009; Yu et al., 2013, 2015, 2017b; Semenova et al., 2016; Masoliver et al., 2017; Marhl and Gosak, 2019).

Neurons communicate mainly by two modalities of synaptic transmission, that is, chemical and electrical synapses (Pereda, 2014; Alcamí and Pereda, 2019), as well as by other means of communication, such as autaptic connections (Bacci and Huguenard, 2006) or presumably *via* magnetic fields (Guo S. et al., 2017; Ma and Tang, 2017). In a plethora of previous theoretical and experimental studies, it has been studied how the interplay between electrical and chemical coupling (Yilmaz et al., 2013; Yu et al., 2020), autapses (Wang et al., 2014; Uzun et al., 2017), and electromagnetic induction (Jia et al., 2019) affect the collective neuronal dynamics, including the coherence resonance (Balenzuela and García-Ojalvo, 2005; Yilmaz et al., 2016; Liu and Yang, 2018; Jia et al., 2021). Moreover, in recent years the research interest is shifting toward neuronal networks composed of two populations, one excitatory and the other inhibitory. In such networks neuronal communication can be of either excitatory or inhibitory nature, and they have been often used as simplified models of local networks in neocortex, hippocampus, as well as other structures (Brea et al., 2009; Isaacson and Scanziani, 2011; Hahn et al., 2019). Most importantly, several studies have shown that inhibition is not only responsible for diminishing neural activity but also leads to complex dynamical patterns that are inaccessible in systems with purely excitatory connectivity (Assisi et al., 2005; Stefanescu and Jirsa, 2008; Ledoux and Brunel, 2011; Bittner et al., 2017; Kim and Lim, 2017; Mongillo et al., 2018; Zhang and Liu, 2019; Rich et al., 2020; Xu et al., 2021). Along these lines, particular attention was given to the interplay between neuronal noise and the excitatory/inhibitory balance. Namely, the presence of inhibitory neurons generates additional nonlinear effects, which can lead to a rich variety of coherent network dynamics that emerges from noisy perturbations in a non-monotonous way (Li et al., 2009; Kawaguchi et al., 2011; Kim et al., 2015; Sancristóbal et al., 2016) and that a fine excitation-inhibition balance can improve the response of the neuronal network (Wang et al., 2012; Guo D. et al., 2017; Yu et al., 2018).

Due to the inherently compound dynamics and multiple facets of interactions that characterize neuronal assemblies, the standard network approach focusing on single networks in isolation might be insufficient to assess the underlying complex activity patterns. Recently, the multilayer network formalism has emerged as a new research direction to engage with such multidimensional systems (Boccaletti et al., 2014; Kivelä et al., 2014; Gosak et al., 2018; Battiston et al., 2020; Torres et al., 2021), including in the field of neuroscience (Bassett and Sporns, 2017; De Domenico, 2017; Maertens et al., 2021). In the context of neuronal networks, different types of interactions can be represented as a multiplex network, where the nodes in all layers are the same, whereas the connections in each layer signify different means of intercellular communication (Gosak et al., 2015; Nicosia and Latora, 2015; Nicosia et al., 2017). These concepts are gaining popularity in the field of computational neuroscience as multiplexing and different forms of interactions between layers were reported to evoke a rich variety of collective phenomena (Nicosia et al., 2013; Jalan and Singh, 2016; Majhi et al., 2017; Ge et al., 2018; Rakshit et al., 2018; Kundu et al., 2019; Parastesh et al., 2019; Sawicki et al., 2019; Bahramian et al., 2021; Kumar Verma and Ambika, 2021). As another option, the multilayer formalism can be used to characterize interactions between different neuronal subpopulations (Andreev et al., 2021) or to characterize heterologous interactions between neurons and other cell types (Virkar et al., 2016; Maertens et al., 2021). In this case, multilayered neuronal interaction schemes represent interdependent networks. Some recent computational studies have focused explicitly on the constructive role of noise in multilayered neuronal networks. It has been shown that multiplexing can give rise to coherence resonance in a two-layered network of excitable neurons (Semenova and Zakharova, 2018). Moreover, the coherence resonance and self-induced stochastic resonance in a multiplex neuronal network can be controlled by the network topology as well as by the intra- and interlayer time-delayed couplings (Yamakou and Jost, 2019; Yamakou et al., 2020). In a specific scenario where only one layer displays noise-induced spiking activity, a weak coupling between two neuronal populations in a multiplexed configuration was found to lead to coherence, anticoherence, and inverse stochastic resonances, as specified by the characteristics of interlayer links (Masoliver et al., 2021).

In this study, we make use of the multilayer network formalism to examine the coherence resonance phenomenon in populations of excitatory and inhibitory neurons. To that purpose, we represent this neuronal population as a two-layered network, where one layer contains inhibitory neurons, the other excitatory neurons, and the interlayer connections represent heterotypic interactions. This framework enables us to systematically investigate how different neurophysiological determinants influence the coherence resonance. Specifically, we investigate how the proportion of inhibitory neurons, the proportion of excitatory interlayer axons, and the architecture of interlayer connections between inhibitory and excitatory neurons affect the nature of the noise-induced dynamics.

## Computational Models and Methods

We study a two-layered network of FitzHugh-Nagumo neurons in the excitable regime with noise. The equations that describe the neuronal dynamics of the *i*th neuron are as follows:

(1) $$\begin{array}{r} \frac{dV_{i}}{dt}=c\left(V_{i}-\frac{V_{i}^{3}}{3}-w_{i}\right)+D\eta \end{array}$$

(2) $$\begin{array}{r} \frac{dw_{i}}{dt}=\frac{1}{c}\left(V_{i}-bw_{i}+a\right) \end{array}$$

where *V*_*i*_ mimics the membrane potential and *w*_*i*_ the recovery variable of the *i*th neuron, *a*, *b*, and *c* are constant parameters related to neuron properties, chosen to be *a* = 0.8, *b* = 0.9, and *c* = 4.5. The last term in Equation 1 represents noise, whereby *D* is noise intensity and η stands for Gaussian white noise with zero mean and unit variance.

**Figure 1:**
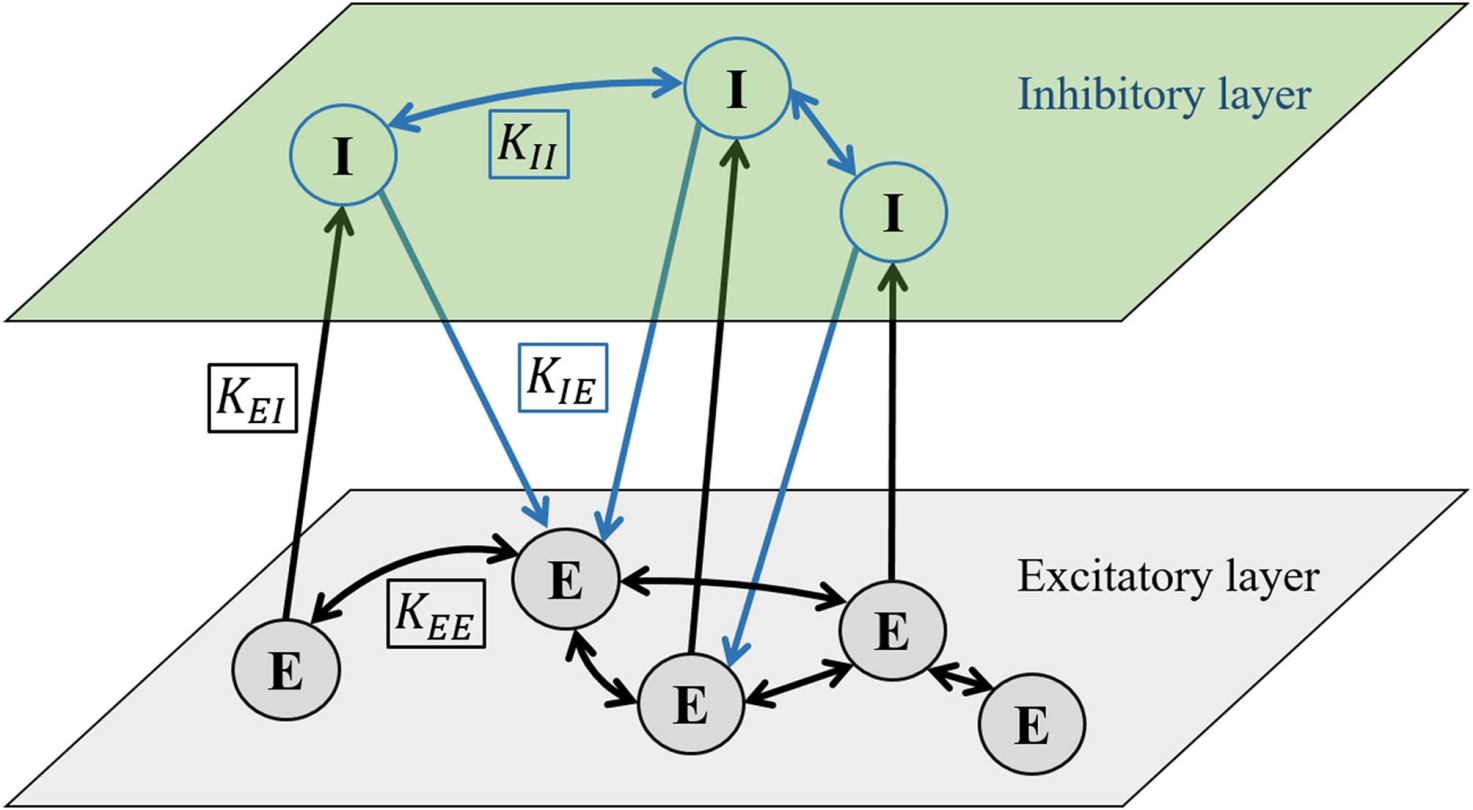
Schematic diagram showing the two-layered network formalism to model the interactions between excitatory (E) and inhibitory (I) neurons. The upper layer is populated by inhibitory neurons and the lower layer by excitatory neurons. Intralayer connections between excitatory neurons *K*_*EE*_ and *K*_*II*_ are bidirectional, whereas the interlayer connections *K*_*EI*_ and *K*_*IE*_ between different neuron types are directed. The directionality of interlayer connections is determined by the parameter ξ, which indicates the probability that the connection will be directed from the excitatory to the inhibitory layer (see main text for details).

We use a multilayer representation of the neuronal population so that one layer is populated by excitatory neurons and the other with inhibitory neurons. Formally, interactions between neurons within the same layer are portrayed by homotypic intralayer connections, whereas the coupling between different types of neurons is characterized by heterotypic interlayer connections. To model interactions between individual neurons, we introduce four different coupling strength coefficients: *K*_*EE*_, *K*_*EI*_, *K*_*IE*_, and *K*_*II*_. First and second indices refer to the type of presynaptic and postsynaptic neuron, respectively (*E*, excitatory and *I*, inhibitory), as schematically presented in Figure 1. We set the standard value of all coupling coefficients to be 0.2. We add coupling terms to Equation 1 and use different coupling coefficients depending on the type of postsynaptic neuron:

(3) $$\begin{array}{r} \frac{dV_{i}}{dt}=c\left(V_{i}-\frac{V_{i}^{3}}{3}-w_{i}\right)+D\eta +K_{EE}V_{i}^{E}-K_{IE}V_{i}^{I} \end{array}$$

for excitatory neurons, and

(4) $$\begin{array}{r} \frac{dV_{i}}{dt}=c\left(V_{i}-\frac{V_{i}^{3}}{3}-w_{i}\right)+D\eta +K_{EI}V_{i}^{E}-K_{II}V_{i}^{I} \end{array}$$

for inhibitory neurons. Equation 2 remains the same for all neurons. $V_{i}^{E}$ and $V_{i}^{I}$ represent sums of all stimuli that the *i*th neuron receives from all coupled excitatory and inhibitory neurons, respectively, and are calculated as follows:

(5) $$\begin{array}{r} V_{i}^{E}=\overset{N_{E}}{\underset{j=1}{\sum }}M_{ji}\left(V_{j}-V_{i}\right), \end{array}$$

(6) $$\begin{array}{r} V_{i}^{I}=\overset{N}{\underset{j=N_{E}+1}{\sum }}M_{ji}\left(V_{j}-V_{i}\right), \end{array}$$

where *N* is the number of all neurons and *N*_*E*_ the number of excitatory neurons. *M*_*ji*_ is the element of the binary coupling matrix *M*, which indicates a connection between the *j*th and *i*th neuron. Indices with *i* ≤ *N*_*E*_ are assigned to excitatory neurons and the rest to inhibitory neurons. The algorithm to generate the two-layered neuronal network composed of homotypic and heterotypic interneuronal connections is explained in more detail in continuation.

Before constructing a two-layered network, we define a parameter γ_*I*_, which describes the proportion of inhibitory neurons so that the number of inhibitory and excitatory neurons (*N*_*I*_ and *N*_*E*_) can be expressed as

(7) $$\begin{array}{r} N_{I}=N\gamma_{I} \end{array}$$

and

(8) $$\begin{array}{r} N_{E}=N\left(1-\gamma_{I}\right)=N-N_{I} \end{array}$$

In our calculations, the number of neurons is set at *N* = 200. Both layers are populated with the proper number of neurons given a particular value of γ_*I*_. We set the standard fraction of inhibitory neurons to γ_*I*_ = 0.2 since inhibitory neurons were found to be less common inside a typical cortex volume compared to excitatory neurons. The positions of neurons in the *xy*-plane are assigned randomly between 0 and 1, with the lower- and upper-layer harboring excitatory and inhibitory neurons, respectively. Connections within the same layer are modeled as a random geometric network where two neurons are connected bidirectionally if the distance between them is less than *R*_*th*_. We determine the value of *R*_*th*_ such that in an average network with γ_*I*_ = 0.1 the average value of intralayer connections per neuron is *k* = 8.0. We find the appropriate value as *R*_*th*_ = 0.126. The same value is chosen for both layers so that the standard values lead to more sparsely connected neurons in the inhibitory layer, as is the case in realistic neuronal assemblies (Börgers and Kopell, 2003).

**Figure 2:**
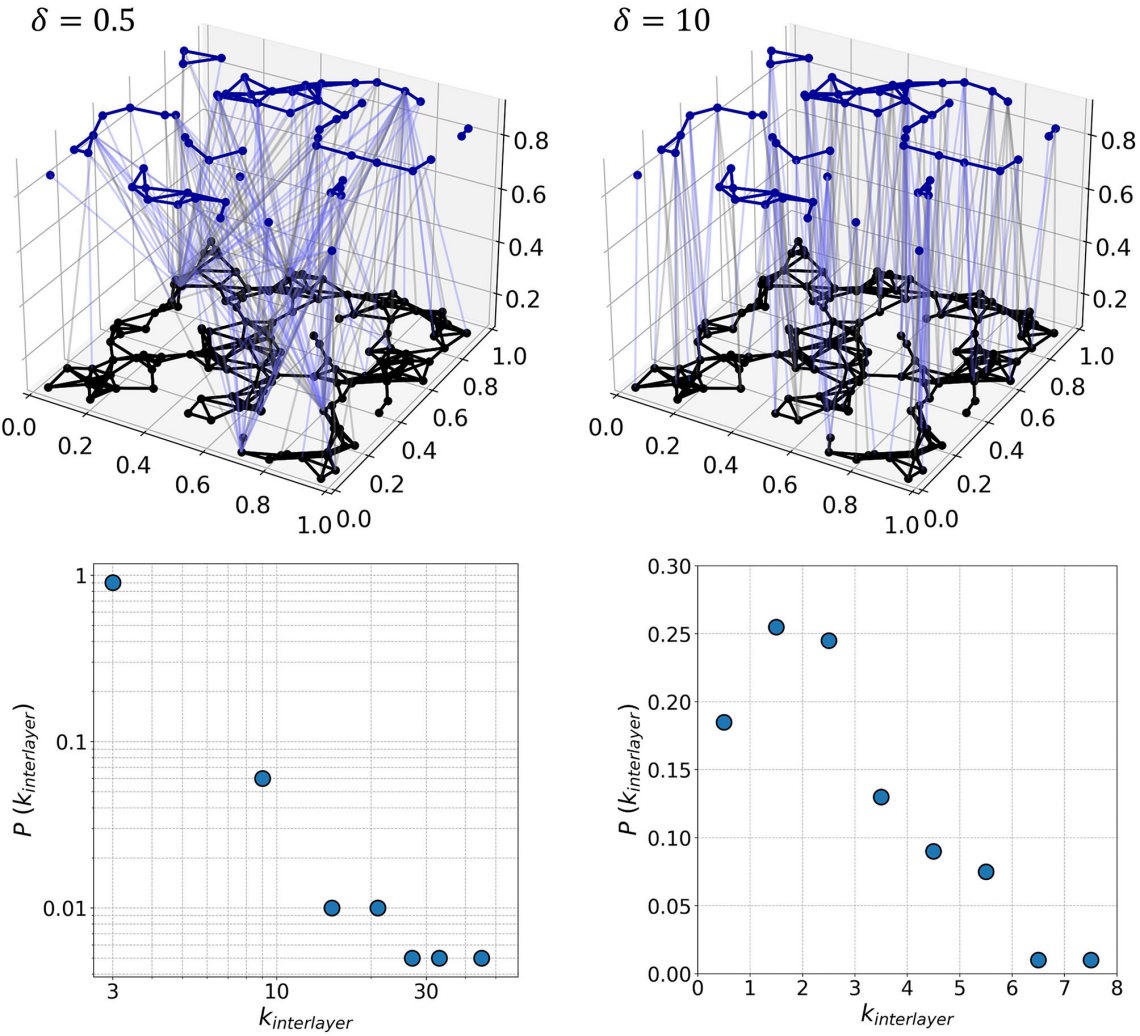
Two-layered network representation of neuronal communication patterns in populations with excitatory (black; lower layer) and inhibitory (upper layer, blue) neurons for two values of the interlayer connectivity parameters δ (upper row) and the corresponding degree distributions of interlayer connections (lower row). For δ = 0.5, there are many long-range connections between both layers and the architecture is very heterogeneous, as indicated by the scale-free character of the interlayer node degree distribution. In contrast, for δ = 10 the distribution of the interlayer excitatory and inhibitory axons is more homogeneous, and the corresponding degree distribution obeys a Poisson distribution. The network consists of 200 neurons, of which 30% were inhibitory (γ_*I*_ = 0.3). The directionality of interlayer connections was assigned at random, as specified by the parameter ξ, which was set at the standard value ξ = 0.5.

To construct interlayer connections, we made use of the vertex fitness network model (Caldarelli et al., 2002; Morita, 2006). Each neuron is assigned a fitness *f*_*i*_ as follows:

(9) $$\begin{array}{r} f_{i}={\left(\frac{i}{N}\right)}^{\frac{1}{1-\beta }} \end{array}$$

where β defines the slope of potential fitness distribution and was set to β = 2.5. After assigning fitnesses to neurons deterministically based on their indices, the fitness values are randomly shuffled between neurons to ensure a well-mixed arrangement across both layers. Whether a connection between an *i*th neuron from the excitatory layer and *j*th neuron from the inhibitory layer exists is determined by the following condition:

(10) $$\begin{array}{r} \Theta <\frac{f_{i}f_{j}}{L_{ij}^{\delta }} \end{array}$$

where *L*_*ij*_ is the distance between neurons in the *xy*-plane, Θ a connectivity threshold, and δ a network parameter that determines the nature of interlayer connections. Small values of δ lead to heterogeneous distribution of interlayer connections, while larger values prefer short-range connections, making the interlayer connectivity more homogeneous. This model represents an alternative to the preferential attachment mechanism (Barabási and Albert, 1999) as it does not rely on network growth and the links are established solely on the assigned fitness values. It should be noted that in our study no correlation between the node importance and dynamical characteristics of neurons was assumed, although this might have been a valid upgrade for heterogeneous neuronal populations. The total number of interlayer connections depends on the value of Θ. We set the average number of interlayer connections per neuron to be ${\bar{k}}_{I}$ = 2.0 and iteratively adjust the threshold value accordingly for each generated network. All interlayer connections are directed whereby their directions are set randomly, as defined by the parameter ξ, which describes the proportion of excitatory interlayer axons or the probability that a given connection will be directed from excitatory to inhibitory layer. For each pair of excitatory and inhibitory neurons that satisfies the connectivity condition (Equation 10), we generate a random number *r* between 0 and 1. If *r* ≤ ξ, the connection is directed toward inhibitory layer representing an excitatory axon coupled to an inhibitory neuron, whereas if *r* > ξ the connection is directed toward the excitatory layer representing an inhibitory axon coupled to an excitatory neuron. We chose the standard fraction of excitatory interlayer axons to be ξ = 0.5 such that both directionalities are equally common. In Figure 2, we show two representative multilayer networks generated at two different values of δ, along with the corresponding interlayer node degree distributions. Evidently, for δ = 0.5, the arrangement of interlayer connections is very heterogeneous, as characterized by a scale-free-like degree distribution. In contrast, for δ = 10 the interlayer connections are established only between neurons that are adjacent in the *xy*-plane and the corresponding degree distribution follows a Poissonian distribution, indicating thereby a homogeneous interaction pattern between both layers.

To determine the amount of coherence in the dynamics of the neuronal population, we computed the autocorrelation function for each neuron (Pikovsky and Kurths, 1997):

(11) $$\begin{array}{r} C_{i}\left(t\right)=\frac{\langle {\tilde{V}}_{i}\left(t\right)\;{\tilde{V}}_{i}\left(t+\tau \right)\rangle }{\langle V_{i}^{2}\rangle -{\langle V_{i}\rangle }^{2}} \end{array}$$

where τ is time lag and $\tilde{V}$_*i*_ deviation of membrane potential *V*_*i*_ from the temporal mean 〈*V*_*i*_〉 at a particular time: $\tilde{V}$_*i*_ = *V*_*i*_ − 〈*V*_*i*_〉. We qualify the coherence of an *i*th neuron with characteristic correlation time *T*_*i*_, defined as (Pikovsky and Kurths, 1997),

(12) $$\begin{array}{r} T_{i}=\int C^{2}\left(\tau \right)d\tau \end{array}$$

We then calculated the average values of *T* for all neurons $\bar{T}$, excitatory neurons ${\bar{T}}_{E}$, and inhibitory neurons ${\bar{T}}_{I}$ accordingly:

(13) $$\begin{array}{r} \bar{T}=\frac{1}{N}\overset{N}{\underset{i=1}{\sum }}T_{i}\;,\;{\bar{T}}_{E}=\frac{1}{N_{E}}\overset{N_{E}}{\underset{i=1}{\sum }}T_{i}\;,\;{\bar{T}}_{I}=\frac{1}{N_{I}}\overset{N}{\underset{i=N_{E}+1}{\sum }}T_{i} \end{array}$$

Higher values of correlation times indicate a higher degree of periodicity in the autocorrelation functions, which signifies a more coherent neuronal activity. In our simulations, the quantification of the coherence for a given set of parameters was computed on the basis of the average of $\bar{T}$, ${\bar{T}}_{E}$, and ${\bar{T}}_{I}$ across 50 independent realizations in order to reduce fluctuations due to stochastic dynamics and from randomness incorporated in network constructions.

## Results

**Figure 3:**
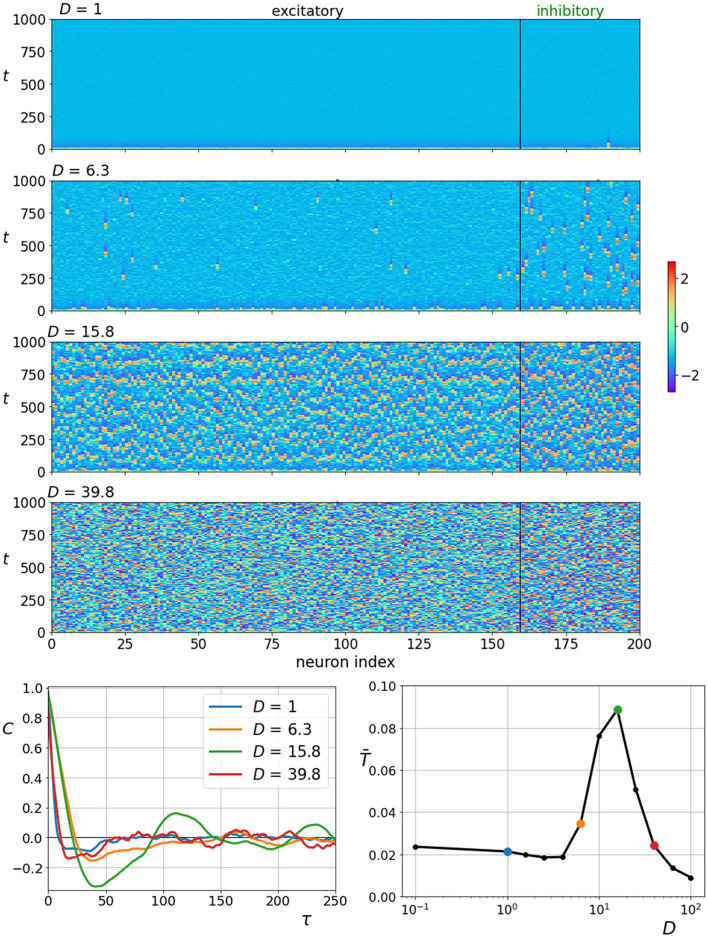
Raster plots of neuronal activity at different noise intensities *D* (upper four panels) with corresponding autocorrelation function plots of an individual neuron (bottom left panel). Average characteristic correlation time $\overset{¯}{T}$ as a function of noise intensity *D* (bottom right panel). Colored points on the resonance curve represent specific noise intensities for which raster plots and autocorrelation plots are shown. Most coherent spiking is observed at around *D* = 15.

First, we examine how coherence resonance manifests itself in the two-layered network composed of excitatory and inhibitory neurons. In the upper four panels of Figure 3, we show space-time plots of neuronal activity obtained at different noise intensities. It can be observed that the behavior reflects typical hallmarks of coherence resonance. Low noise intensity evokes seldom and irregular firings, high noise intensity leads to an erratic activity, whereas intermediate values of noise evoke the most coherent spatiotemporal patterns of neuronal dynamics. To quantify the observed behavior, we computed the normalized autocorrelation function (Equation 11) and the corresponding correlation times (Equation 12). The results in the lower row of Figure 3 clearly demonstrate that the stronger the periodic components of the autocorrelation function and the correlation times are the highest at intermediate noise intensities, corroborating thereby the existence of coherence resonance. In this simulation, we used the standard values of all parameters, namely, *D*, γ_*I*_, ξ, *K*_*EE*_, *K*_*II*_, *K*_*EI*_, *K*_*IE*_, and δ. In what follows, we will investigate how variations of those parameters that refer principally to the characteristics of the multilayer neuronal network affect the noise-induced dynamics and the collective response of the system.

**Figure 4:**
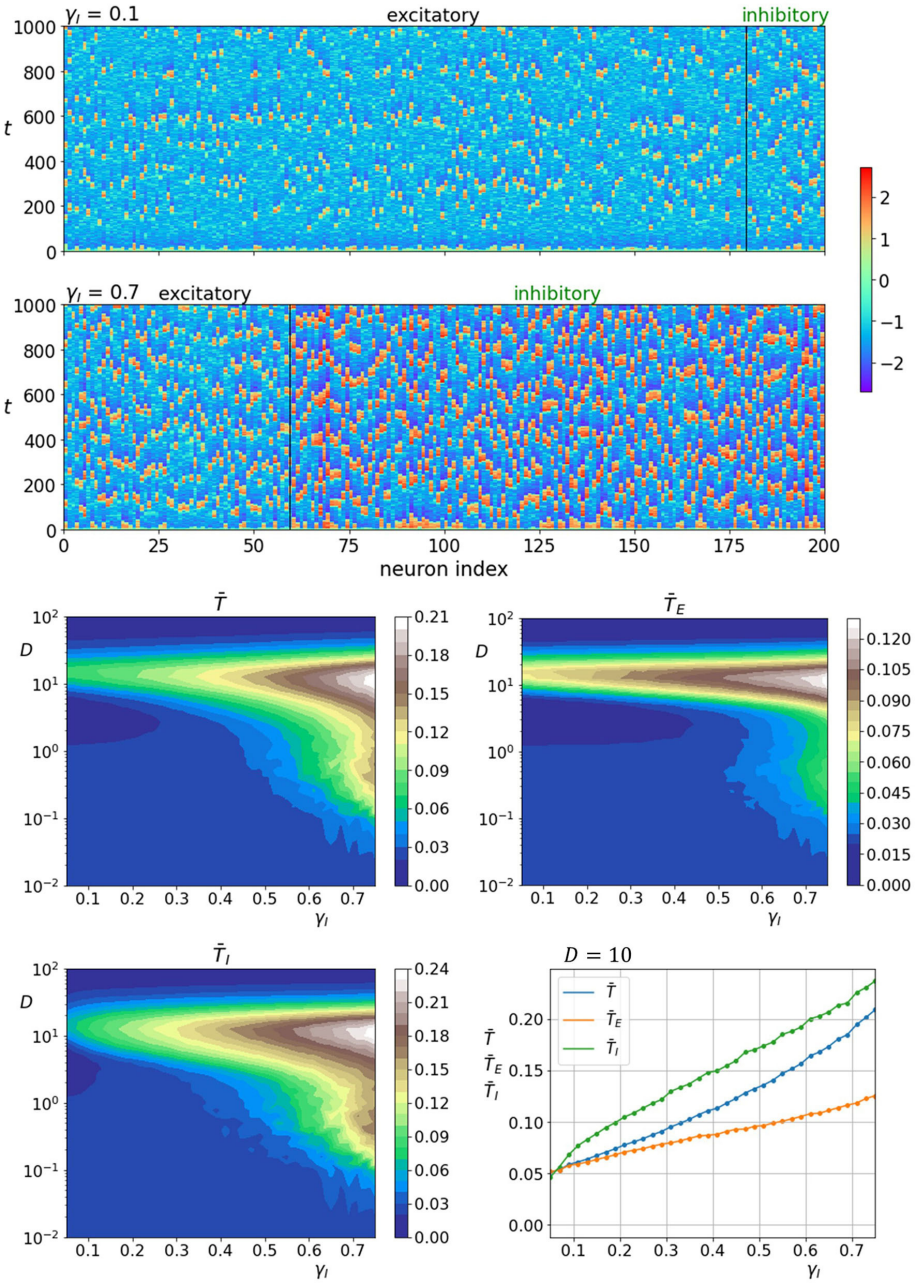
Raster plots of neuronal activity for two different proportions of inhibitory neurons γ_*I*_ (upper two panels). Quantification of the coherence resonance phenomenon in dependence on the noise intensity *D* and the fraction of inhibitory neurons γ_*I*_ (lower four panels). The contour plots show the color-coded average values of correlation times for all neurons $\overset{¯}{T}$, excitatory neurons ${\overset{¯}{T}}_{E}$, and inhibitory neurons ${\overset{¯}{T}}_{I}$. The lowermost panel on the right represents the average correlation times values near the optimal noise intensity values (*D* = 10).

We start by analyzing how the fraction of inhibitory neurons γ_*I*_ influences neuronal dynamics. The upper two panels of Figure 4 show space-time plots of neuronal activity for two different fractions of γ_*I*_. It is apparent that the activity of inhibitory neurons is intrinsically higher and increases further when their proportion is high (γ_*I*_ = 0.7). In addition, the spatiotemporal patterns appear to be more ordered when the number of inhibitory neurons is high. The lower four panels of Figure 4 show the color-coded values of average correlation times in dependence on the fraction of inhibitory neurons as well as noise intensity. It can be seen that increasing the fraction of inhibitory neurons improves the coherence in both layers. Moreover, as the proportion of inhibitory neurons is unrealistically high, the regularity of noise-induced oscillations begins to increase already at lower noise intensities. To visualize the role of the number of inhibitory neurons in further detail, we show in the lowermost panel on the right all correlation times as a function of γ_*I*_ near the optimal noise intensity.

**Figure 5:**
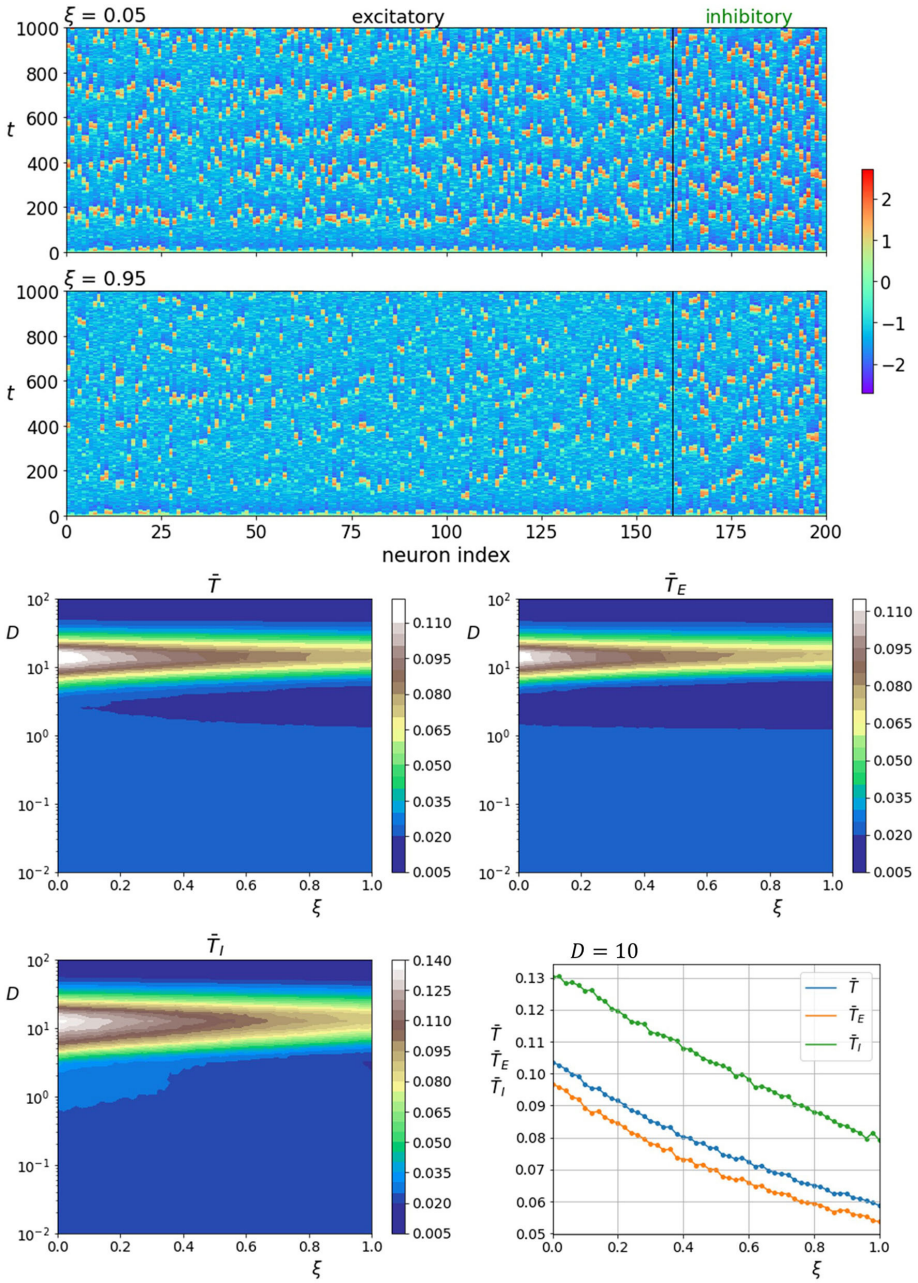
Raster plots of neuronal activity for two different interlayer proportions of excitatory axons ξ (upper two panels). Quantification of the coherence resonance phenomenon in dependence on the noise intensity *D* and the interlayer proportion of excitatory axons ξ (lower four panels). The contour plots show the color-coded average values of correlation times for all neurons $\overset{¯}{T}$, excitatory neurons ${\overset{¯}{T}}_{E}$, and inhibitory neurons ${\overset{¯}{T}}_{I}$. The lowermost panel on the right represents the average correlation times values near the optimal noise intensity values (*D* = 10).

Next, we examine how the nature of the neuronal dynamics depends on the fraction of excitatory interlayer axons ξ. The upper two panels of Figure 5 show space-time plots of neuronal activity for two different values of ξ. Lower fractions of excitatory axons seem to increase the activity in both layers. The same trend is observed for the coherence as seen on the lower four panels of Figure 5 that show average correlation times in dependence on the fraction of excitatory interlayer axons as well as noise intensity. The lowermost right panel showing average correlations time near the optimal noise intensity (*D* = 10) also indicates a similar increase in the coherence of noise-induced oscillation in both layers.

**Figure 6:**
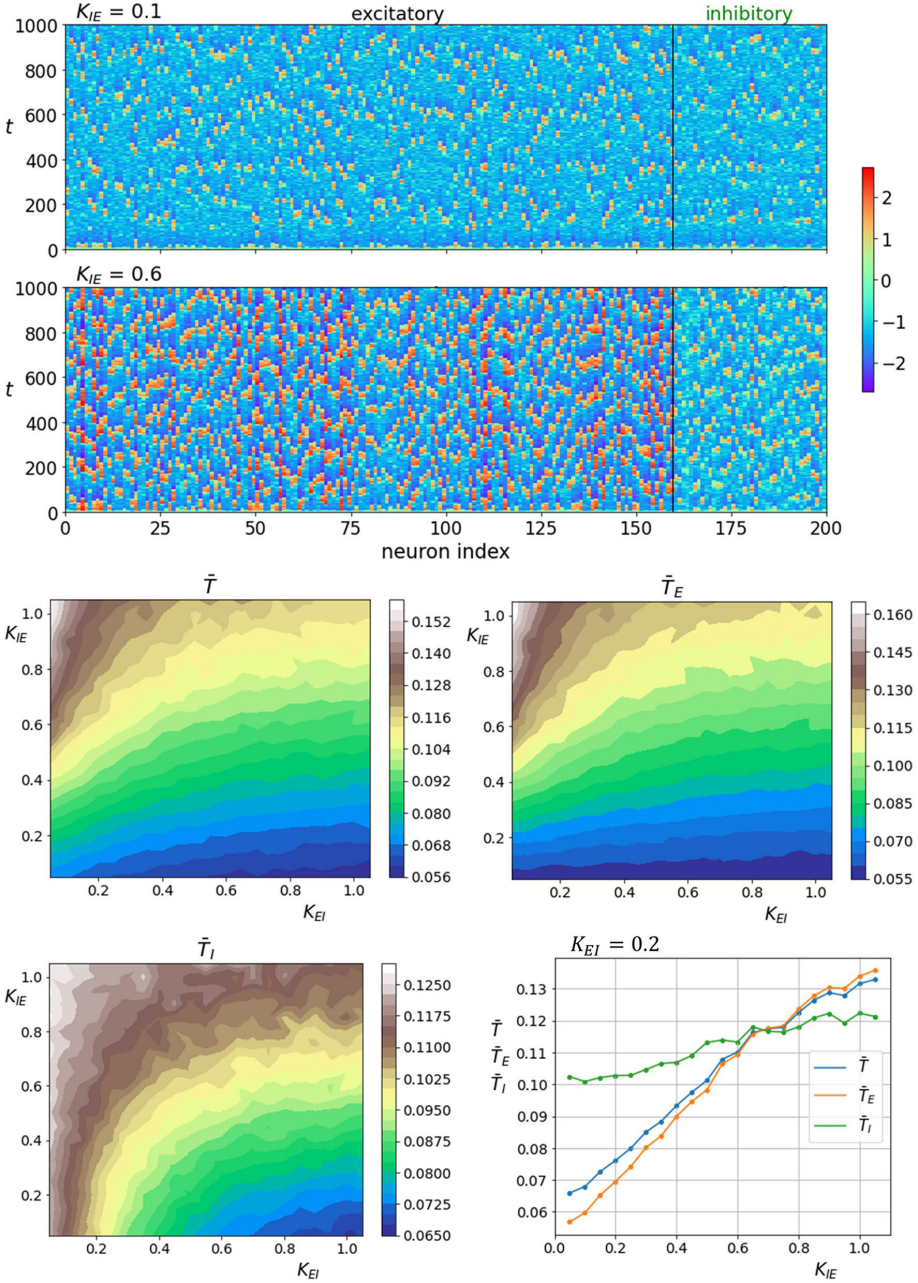
Raster plots of neuronal activity for two different interlayer coupling strengths *K*_*IE*_ where *K*_*EI*_ = 0.2 (upper two panels). Quantification of the coherence resonance phenomenon in dependence on the interlayer coupling strengths *K*_*EI*_ and *K*_*IE*_ (lower four panels). The contour plots show the color-coded average values of correlation times for all neurons $\overset{¯}{T}$, excitatory neurons ${\overset{¯}{T}}_{E}$, and inhibitory neurons ${\overset{¯}{T}}_{I}$. The lowermost panel on the right represents the average correlation times values in dependence on coupling strength *K*_*IE*_ at a constant value of *K*_*EI*_ = 0.2.

We continue with the analysis of interlayer coupling strengths *K*_*EI*_ and *K*_*IE*_. The upper two panels of Figure 6 show space-time plots of neuronal activity for two different coupling strengths of inhibitory interlayer axons *K*_*IE*_, where coupling strength of excitatory interlayer axons is held constant *K*_*EI*_ = 0.2. The lower four panels show average correlation time values as a function of both interlayer coupling strength, whereas the lowermost right panel shows them only in dependence on inhibitory axons coupling strength *K*_*IE*_. We see an obvious increase in regularity when the coupling of inhibitory axons to excitatory layer gets stronger. Notably, the increase in coherence affects the excitatory layer more profoundly as the relative gain in average correlation time in the inhibitory layer is significantly lower. In contrast, enhancing the coupling strength of excitatory interlayer axons decreases the level of regularity in both layers.

**Figure 7:**
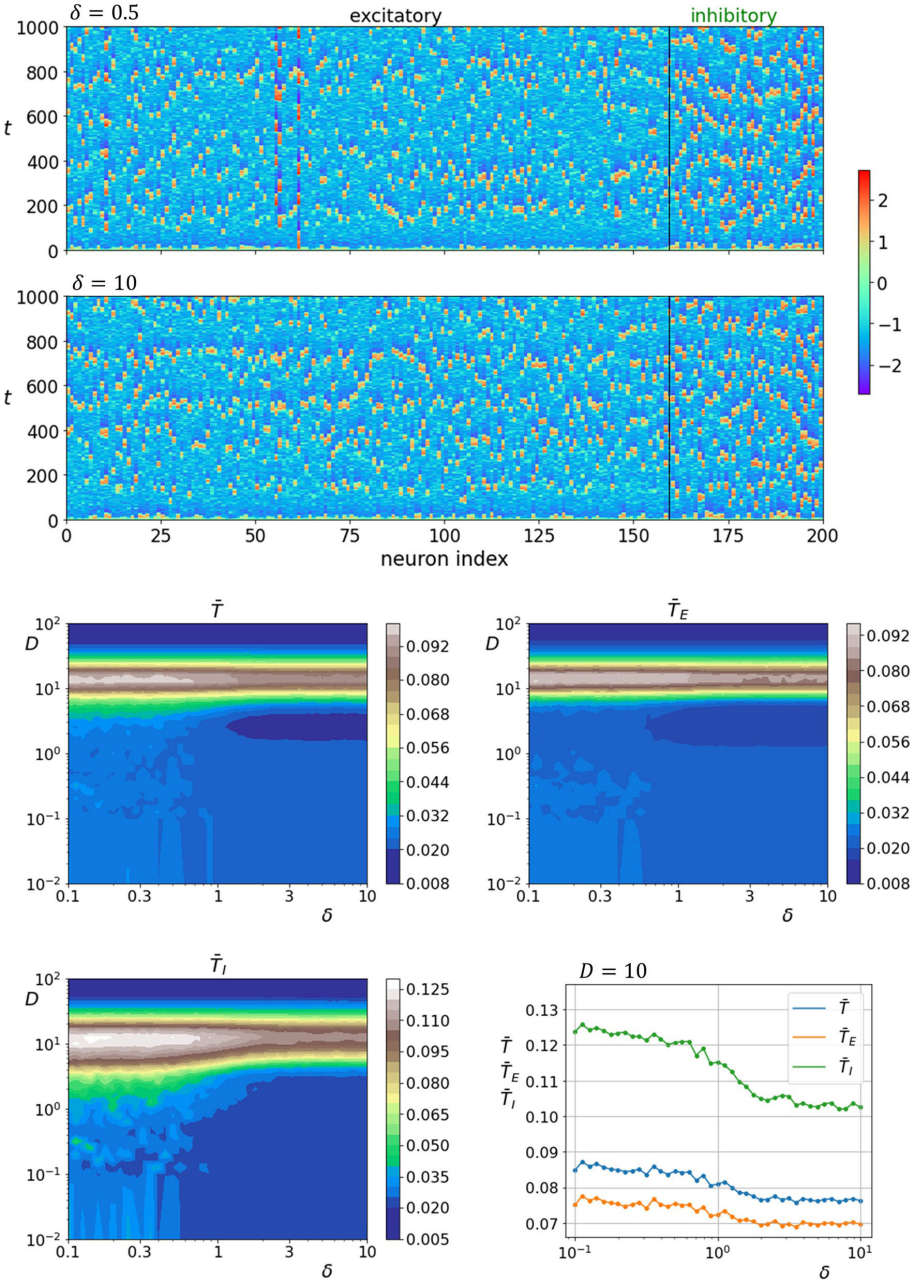
Raster plots of neuronal activity for two different values of parameter δ (upper two panels). Quantification of the coherence resonance phenomenon in dependence on the noise intensity *D* and parameter δ (lower four panels). The contour plots show the color-coded average values of correlation times for all neurons $\overset{¯}{T}$, excitatory neurons ${\overset{¯}{T}}_{E}$, and inhibitory neurons ${\overset{¯}{T}}_{I}$. The lowermost panel on the right represents the average correlation times values near the optimal noise intensity values (*D* = 10).

Finally, we investigate how neuronal activity is affected by the interlayer network parameter δ. The upper two panels of Figure 7 show space-time plots of neuronal activity for two different values of parameter δ. The lower four panels show average correlation times in dependence on parameter δ and noise intensity, whereas the lowermost panel on the right shows the relation between the correlation times and interlayer network parameter δ near optimal noise intensity. It can be seen that varying the parameter δ affects the noise-induced neuronal dynamics so that a higher degree of regularity is attained when the interlayer connectivity is heterogeneous (δ < 1). The effect is slightly more pronounced for the inhibitory layer. These results in combination with previous reported findings seem to point toward a general pattern where the overall coherence of neuronal dynamics can be enhanced by a stronger damping of the excitatory layer. This can be achieved by either a larger fraction of inhibitory neurons, larger fraction of inhibitory interlayer axons, a stronger coupling of inhibitory axons with excitatory layer, or a more efficient interaction patterns between both types of neurons.

## Discussion

Coherent neuronal activity is a putative mechanism whereby neuronal populations subserving specific functions communicate for the purpose of establishing dynamical patterns that accomplish perception, cognition, and action (Riehle et al., 1997; Oram et al., 1999; Horwitz and Braun, 2004). It is nowadays a well-established fact that the creation of the neuronal dynamics relies significantly on random fluctuations (Stein et al., 2005; Faisal et al., 2008; Chialvo, 2010; McDonnell and Ward, 2011; Guo et al., 2018). Previous research encompassing tissue slice preparations, whole brains, and computational models has revealed that neural synchronization can be facilitated by the addition of optimal amounts of neuronal noise, i.e., neurons can exploit noise to enhance the regularity of their pulsing dynamics. The numerous experimental evidence showing the constructive role of noise at the microscopic (Douglass et al., 1993; Gluckman et al., 1996; Gu et al., 2002; Manjarrez et al., 2002; Ward et al., 2010; Sancristóbal et al., 2016) and macroscopic (Collins et al., 1996; Simonotto et al., 1997; Russell et al., 1999; Hidaka et al., 2000; Kitajo et al., 2003; Itzcovich et al., 2017) levels of neuronal organization has evoked immense interest of computational neuroscientists to investigate the underlying mechanisms and functional implications (Lindner et al., 2004; Sagués et al., 2007; McDonnell and Abbott, 2009; Calim et al., 2021). In recent years, the models are becoming increasingly comprehensive as they incorporate several types of neuronal populations, different types of interactions, information transmission delays, plasticity in connectivity patterns, etc. Moreover, along with the developments in the field of network science, the scope is shifting to multilayer networks as this novel concept offers a more comprehensive framework to assess the complex interactions across multiple facets of neurophysiological relationships and the resulting dynamical phenomena (Boccaletti et al., 2014; Kivelä et al., 2014; Gosak et al., 2018, 2022; Battiston et al., 2020; Torres et al., 2021).

In this study, we aimed to extend the scope of coherence resonance in a multilayer network model of neuronal dynamics. We considered a mixed heterogeneous and excitable neural population composed of excitatory and inhibitory neurons. The multilayer network formalism was used to represent these subpopulations so that each neuron type occupied a separate layer and the directed interlayer connections represented excitatory or inhibitory axons. This setting enabled us to systematically investigate how different neurophysiological determinants affected the characteristics of the noise-driven neuronal dynamics. We focused specifically on the proportions of inhibitory neurons and excitatory interlayer axons as well as on the strength and structure of the interlayer connectivity. Our numerical calculations have revealed that the proposed setup represents a viable route for the realization of coherence resonance (Figure 3). The resonant behavior was found to be quite immensely affected by the number of inhibitory neurons so that a higher fraction of inhibitory units enhanced the regularity of the entire system (Figure 4). Moreover, a higher fraction of excitatory axons was found to reduce the regularity of noise-induced oscillations (Figure 5). The accuracy of neuronal excitations was also found to be affected by the interplay between the coupling strength between the excitatory in inhibitory layer, whereby the coherence resonance was more pronounced when the influence of the inhibitory layer was promoted. Interestingly, the regularity in the excitatory layers turned out to be affected more (Figure 6). Finally, we investigated how neuronal activity depends on the structure of the heterotypic interactions between the excitatory and inhibitory neurons. By varying the parameter for interlayer connectivity, the structure of connections between both layers was smoothly altered between a highly heterogeneous scale-free-like structure and a rather homogeneous organization with no substantial differences in the number of connections between individual neurons (Figure 2). It turned out that the more heterogeneous configuration evoked a higher degree of regularity in the neuronal dynamics (Figure 7). Apparently, the more heterogeneous and scale-free-like interlayer connectivity structure represents a more efficient setting for information transmission as the homogeneous interlayer connectivity pattern (Vragović et al., 2005) and as such enhances the communication between both layers, which makes the influence of the inhibitory neurons more pronounced. To sum up, our findings indicate that giving prominence to the inhibitory layer, either by increasing the number of inhibitory neurons or enhancing the coupling strength or efficiency from the inhibitory to the excitatory layer, always acts as a promoter of regular neuronal activity.

Previous computational studies have already investigated collective activity in networks of interconnected excitatory and inhibitory neurons as such networks are ubiquitous in the brain (Best et al., 2007; Sukenik et al., 2021). In the last decade, computational models and analyses have played an important role in identifying the nature of neuronal rhythmicity that orientates from the complex interplay between subpopulations of inhibitory and excitatory cells (Jadi and Sejnowski, 2014; Hahn et al., 2019; Sukenik et al., 2021). It has been shown that the presence of inhibitory neurons can facilitate rhythmic activity in neural networks (Shimokawa and Shinomoto, 2006; Cheng and Cao, 2017; Ma and Tang, 2017) and that the presence of heterogeneous inhibitory neurons can optimize their responsiveness to external stimuli (Di Volo and Destexhe, 2021). In particular, the architecture of the neuronal network was reported to play a major role in this respect (Rich et al., 2020).

Furthermore, in the context of stochastic facilitation, it has been reported that fine-tuning of inhibitory synapses can improve frequency-difference-dependent stochastic resonance in neuronal networks (Guo D. et al., 2017) and that the presence of inhibitory chemical synapses at the intralayer levels optimizes stochastic resonance in multiplex neural networks (Yamakou et al., 2020). It has also been shown that the presence of inhibitory neurons can enhance the coherence resonance behavior in neuronal networks, but the effect is rather complex and depends on the interplay between the coupling characteristics and noise (Yu et al., 2018). Along similar lines, inhibitory autapses have been recently recognized as another suppressive agent that can facilitate coherence resonance in neuronal networks (Jia et al., 2021).

## Conclusion

Our results demonstrate another example of how the influence of inhibitory neurons can lead to an improved regularity of the noise-driven neuronal dynamics, which we expand to a multilayer network concept. The proposed computational model enabled us to systematically investigate the role of different neuronal network parameters and allowed us to gain a better understanding on how the architecture of interlayer connections between inhibitory and excitatory neurons affected the nature of neuronal activity patterns, even though future studies will be required to gain further insights into the underlying mechanisms. Furthermore, interesting future research directions on the topic would be to use more realistic neuronal models that could incorporate a detailed description of neuronal heterogeneity, synaptic plasticity, information transmission delays, and signaling *via* autaptic connections. All these features are genuine neurophysiological determinants that have been shown to affect the collective dynamics of neuronal networks. Most importantly, the proposed multilayer network representation of the neuronal subpopulations can serve as a solid ground for further upgrades in this direction.

## Data Availability Statement

The original contributions presented in the study are included in the article/supplementary material, further inquiries can be directed to the corresponding author.

## Author Contributions

DR and MG conceived the research, designed the model, reviewed and edited the manuscript, and gave final approval for publication. DR performed calculations and prepared the figures. MG wrote the initial draft of the manuscript. All authors contributed to the article and approved the submitted version.

## Funding

The authors acknowledge the financial support from the Slovenian Research Agency (grant no. P3-0396, I0-0029, J1-2457, J3-2525, N3-0133, and J3-3077).

## Conflict of Interest

The authors declare that the research was conducted in the absence of any commercial or financial relationships that could be construed as a potential conflict of interest.

## Publisher's Note

All claims expressed in this article are solely those of the authors and do not necessarily represent those of their affiliated organizations, or those of the publisher, the editors and the reviewers. Any product that may be evaluated in this article, or claim that may be made by its manufacturer, is not guaranteed or endorsed by the publisher.
